# Social and health vulnerability of homeless people

**DOI:** 10.1590/1980-220X-REEUSP-2022-0379en

**Published:** 2023-09-15

**Authors:** Leris Salete Bonfanti Haeffner, Dirce Stein Backes, Gina de Souza Castro Hammel, Francisca Georgina Macedo de Sousa, Irani Rupolo, Luciane Najar Smeha

**Affiliations:** 1 Universidade Franciscana, Programa em Saúde Materno-Infantil, Santa Maria, RS, Brazil.; 2 Universidade Federal do Maranhão, Programa de Pós-Graduação de Enfermagem, São Luis, MA, Brazil.; 3 Universidade Franciscana, Santa Maria, RS, Brazil.

**Keywords:** Social Vulnerability, Ill-Housed Persons, Pandemics, Community Health Nursing, Nonlinear Dynamics, Vulnerabilidad Social, Personas con Mala Vivienda, Pandemias, Enfermería en Salud Comunitaria, Dinámicas no Lineales, Vulnerabilidade Social, Pessoas Mal Alojadas, Pandemias, Enfermagem em Saúde Comunitária, Dinâmica não Linear

## Abstract

The objective is to conduct a theoretical reflection on the social and health vulnerability of homeless people, from the perspective of complexity thinking. Study with a theoretical and reflective approach that accessed bibliographical sources of contemporary authors who seek to understand the phenomenon of homeless populations and, at the same time, attribute theoretical support from the reference of complexity, under a critical and analytical bias. Health is conceived as a subsystem of the social system that transcends any linear and punctual diagnostic perspective. Theoretical reflection on the social and health vulnerability of homeless people sparks a unique and multidimensional apprehension of the human being – a complex unit par excellence, which demands equally complex interventions.

## RESUMO

Objetiva-se conduzir reflexão teórica sobre a vulnerabilidade social e de saúde de pessoas em situação de rua, na perspectiva do pensamento da complexidade. Estudo de abordagem teórico-reflexiva que acessou fontes bibliográficas de autores contemporâneos que buscam compreender o fenômeno das populações em situação de rua e, paralelamente, aprofundar a temática à luz do referencial da complexidade, sob um viés crítico e analítico. Concebe-se a saúde como subsistema do sistema social que transcende qualquer perspectiva diagnóstica linear e pontual. A reflexão teórica acerca da vulnerabilidade social e de saúde de pessoas em situação de rua acende uma apreensão singular e multidimensional de ser humano – unidade complexa por excelência, que demanda intervenções, igualmente, complexas.

## RESUMEN

El objetivo es realizar una reflexión teórica sobre la vulnerabilidad social y sanitaria de las personas en situación de calle desde la perspectiva del pensamiento de la complejidad. Se trata de un estudio con enfoque teórico y reflexivo basado en fuentes bibliográficas de autores contemporáneos que buscan comprender el fenómeno de las poblaciones sin hogar y, paralelamente, aportar sustento teórico desde el marco de la complejidad, bajo un sesgo crítico y analítico. La salud está concebida como un subsistema del medio social que trasciende cualquier perspectiva diagnóstica, lineal y puntual. La reflexión teórica sobre la vulnerabilidad social y sanitaria de las personas en situación de calle suscita una percepción singular y multidimensional del ser humano, unidad compleja por excelencia, que exige intervenciones igualmente complejas.

## INTRODUCTION

The present study originated from the analysis of data/indicators on people living on the streets. A study developed by the Institute for Applied Economic Research (IPEA) concludes that in March 2020 more than 221,000 people were living on the streets in Brazil, which represents a 140% increase in this population since 2012^(1)^.

The increase in this population contingent is notorious for those who walk the streets of cities and the questions are almost always the same: why do so many people live on the streets and starve, considering that Brazil is one of the most expressive countries in the production of foods? What is the origin of the mismatch between what is produced and what is actually available to citizens? How to think about health, medical/nursing/psychological diagnosis in the face of the unknown, random and uncertain?

The National Policy for the Homeless Population, within the scope of Brazil, considers the homeless population to be the group of people who have extreme poverty in common, interrupted family ties due to the abusive use of alcohol and drugs and the lack of regular conventional housing, among other aspects^(2)^. These are, therefore, population groups that use public places to maintain, temporarily or permanently, their housing and survival conditions^(3,4)^.

The Covid-19 pandemic has worsened the living and health conditions of people who experience poverty, marginalization, stigmatization and social discrimination, especially with regard to access to food, hygiene and essential human needs. In addition to being exposed to social inequalities, homeless people became even more vulnerable, not only in economic terms, but, above all, due to the loss of their human and social identity^(5,6,7,8)^.

The homeless population is therefore among the groups that most resisted Covid-19 and that will continue to suffer from the new health crises associated with endemic events aggravated by the climate crisis^(9)^. In an attempt to escape, these populations migrate to urban centers where they face poverty, precarious housing situations and, simultaneously, experience violence, rejection and social invisibility. Migration combined with homelessness is, therefore, a multifaceted and complex social phenomenon, as it involves multiple cultures, different people, different processes, flows and experiences^(10,11)^.

The phenomenon of homeless people must be analyzed as a multifactorial and multidimensional social event, not reducible to unemployment or economic factors of production relations, but under the bias of reproduction of social relations that involve such dynamics^(12)^. Health, likewise, must be understood as a dynamic, unique, complex system, interconnected with different social systems that aim to promote healthy living for individuals, families and communities from a socio-ecosystemic perspective^(13)^.

The objective is, based on the above, to conduct a theoretical reflection on the social and health vulnerability of homeless people, from the perspective of complexity thinking.

## METHOD

Study with a theoretical and reflective approach that accessed bibliographic sources of contemporary authors who seek to understand the phenomenon of homeless people and, at the same time, theoretical support in the thought of complexity, under a systemic critical and analytical perspective. The theoretical-complex basis is based on principles that allow expanding and contextualizing the different social phenomena, namely: dialogic, organizational resource, hologramatic^(14)^.

The homeless person is apprehended, in the light of complexity thinking, as a singular and multidimensional subject. Under this impulse, the thought of complexity establishes itself as a relevant reference to the understanding that homeless people expect and need to ensure their human and social dignity.

The complex thought proposed by Edgar Morin^(14–17)^, one of the most respected contemporary thinkers, it enables a methodological path that transcends predefined ideas of apprehension of social phenomena. The researcher, under this thinking, is induced to lead his own path based on his experiences and from his formative path. Complexity requires thinking the universal and the particular in the same movement. Being complex means, from this point of view, defending the importance of the universal and the particular, the general and the singular, the common among men and what differentiates them, in order to recognize that we are all equal in difference.

The methodological path materializes, in this direction, when investigating and weaving together the experiences lived in learning, articulating and promoting health and social well-being. Therefore, health is conceived as a subsystem of the larger social system, based on a diagnostic framework that transcends any linear and punctual diagnostic perspective. Thus, the study is based on theoretical productions that preserve systemic-complex thinking in their genesis, above all, inducing rupture^(18,19)^. In this course, conceptions such as: human being, citizenship and health are explored.

### Social System versus Health System: Necessary Approximations

**Chart 1:**
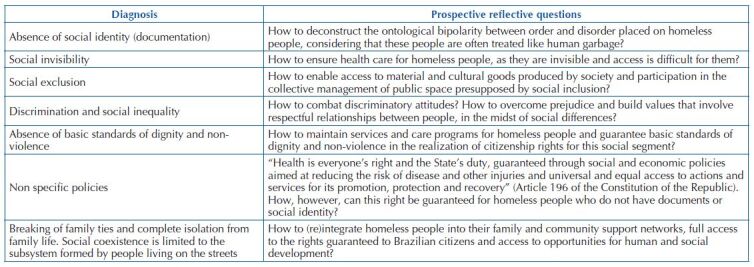
Social diagnosis versus health diagnosis mediated by prospective reflective questions – Santa Maria, RS, Brazil, 2022.

Initially, a reflective framework is presented that transcends any punctual and linear diagnostic perspective and an attempt is made to reveal health as a subsystem of the social system. Health is understood as a social good, a community good, whose concept can only be understood in the light of social determinants and constraints. The aim is to apprehend, however, not the answers and certainties of linear diagnoses, but to launch prospective reflective questions, as shown in Chart 1.

The theoretical-complex discussion, based on the principles of dialogic, organizational and hologramatic resources, will be conducted through the delineation of three exploratory categories: Human being – complex unit; Citizenship for whom and for what? and Health as a social good.

### Human Being – Complex Unit

For Edgar Morin^(15,16)^, it is necessary to differ from reductionist thinking to that which expands and contextualizes. The human being is, par excellence, a being of multiple relations, interactions and systemic associations – a singular, multidimensional, dialogic, complex being. The human being is, therefore, endowed with complexities allied to the phenomena that are imposed by the social, economic and cultural environment in which he is inserted. It is also endowed with senses with an emphasis on imagination and fantasy due to the extraordinary role they play in human consciousness^(20)^.

The human being moves as a social being in evolution, through disruptive and dialogical processes, enhanced by the ability to be singular and multiple, in order to enable new and ever more complex communications. Thus, interactions between individuals produce society, which produces culture and is produced by it, according to the principle of recursion proposed by Morin^(14,15)^.

In this evolutionary social dynamic, the principle of dialogic, which maintains duality in unity, comprises two apparently complementary and antagonistic logics, but necessary for human and social evolution. Dialogic makes it possible to assume the inseparability of contradictory notions in order to conceive of the same complex phenomenon^(14)^. Under this approach, the dialogue between opposing notions can stimulate and promote harmonious and healthy living spaces, without excluding homeless people.

In its multidimensional conception, the human being is not reduced to the physical, racial dimension or to a certain social class. Its nature is relational, associative and evolutionary par excellence. Thus, the homeless person, like any other human being, is a citizen and social being and, by itself, singular and greater than the sum of singularities and differences, capable of building and attributing meanings that produce society^(19)^.

Understanding the human being, a singular subject and member of a society, means singularizing from diversity and transcending to reach the unity of the multiple^(18)^. Under this approach, the health system needs to consider the human being in his dynamics of living healthy, in the light of social determinants and constraints^(13)^.

Morin argues that it is necessary to teach human understanding based on transdisciplinarity and the contextualization of reality, in the sense of integrating and interconnecting what is separated. The living systems that make up society are in permanent evolutionary movement, either by order or by disorder, which produces prospective changes. Under this approach, Morin^(16)^defends an integral and contextualized thought, that is, a non-mutilating thought that leads to punctual, reducing and excluding decisions.

From this perspective, how to apprehend and welcome the human being – homeless person – as a relational and social being, part of an evolving and civilized community? Achieving a systemic-complex thinking in the social and health context implies integrating what is dispersed and bringing together people and social groups that appear, in society, as subjects without name and identity.

### Citizenship, for Whom and for What?

The term citizenship originates from the Latin civitas, which means city. Citizen is, therefore, the one who integrates and inhabits the city, based on civil, political and social rights that develop from the idea of what is best for the social group. Therefore, citizenship confers and ensures rights, in addition to the duty to fight for them. Citizenship also represents the need to recognize new rights^(21,22)^.

Citizenship is a topic discussed and evolving in different social contexts. Study shows that the loss of citizenship of a person or social group represents the loss of their identity, their value and their rights^(23)^. Products and social phenomena are, in the light of complex thinking, the cause that produce them, just as society is the product of interactions and evolutionary associations between people^(14,15)^. It is through interactions that social people, in general, develop, approach and coexist in view of the common good.

The phenomenon – homeless people is, by itself, a complex phenomenon, a humanitarian disorder, which requires human solidarity, welcome and unique understanding not of a stranger, but of a citizen human being who shares the same rights and duties as citizenship. The homeless person does not want to be seen, remembered and supported as an anonymous object, but wants to be recognized by his name, identity, family and cultural history and origin. Wants to be seen and welcomed as a human being who integrates and inhabits the city in the logic of the organizational resource principle – unity in diversity, as well as diversity in unity^(24,25)^.

Recognizing the human being as a unique, multidimensional subject and citizen is, therefore, essential for achieving more resolute and prospective results. Respecting human uniqueness, each subject and social citizen will always be greater than the sum of the individual parts. To apprehend the human being as a citizen is to integrate him and place him in the Universe^(26)^. Every individual, from the most restricted to the most banal of lives, constitutes himself in a cosmos that, through family interactions and established exchanges between broader systems, such as the community and school, becomes able to grasp the human condition and the exercise of citizenship^(19)^.

The human being produces society in interactions and through interactions, but society, as it emerges, produces the humanity of these individuals, providing them with language and culture. This process will have repercussions on the individual and, consequently, on their healthy living. These interactions and associations “modify the behavior or nature of elements, bodies, objects and phenomena, which influence and are influenced by”^(15)^.

What is the concept of citizenship, however, for homeless people? How to think of strategies that contribute to the promotion of citizenship and the well-being of homeless people – excluded from any expression and social manifestation? The thought of complexity is not reduced, under this approach, to storing knowledge, but to developing the integrative and associative ability of subjects, apparently, devoid of the principle of citizenship and attributing to them a meaning of life, of living healthy and of well-being^(18,19)^.

### Health as a Social Good

Health involves movements that recreate the dynamics of life amid the notions of order and disorder. In this relationship, healthy living implies developing systemic-evolutionary pathways capable of transcending fragmentation, the biomedical logic and the medicalization of the health-disease process^(12)^.

It was evident, from the comings and goings through the streets of the city, that people on the streets do not only feel the lack of work, food, shelter, warmth, but, above all, they lack respect, dignity and someone who listens to them and accepts their needs, anxieties, fears and insecurities. This thinking was translated into a statement published in a local newspaper: “I don’t just want people to stop to give me a coin or food. I want people to stop and listen to what I have to say.”

Therefore, what is the meaning of health and healthy living for homeless people? What does health and healthy living mean for health professionals and how to conduct dialogic and listening processes with homeless people? Based on the previously expressed testimony, health transcends the biological bias and punctual and linear diagnoses that apprehend the human being, only in his apparent vulnerabilities. Understood in their uniqueness, the human being is capable of attributing meanings to life, health, citizenship, to the extent that he is heard and welcomed in his needs.

The hologramatic principle conceives, in this sense, the part in the whole and the whole as being greater than the sum of the parts. “This idea transcends reductionisms that only see parts or holism that only sees the whole”. Systemic-complex interactivity implies recursive movements in which the whole and the part interact and evolve simultaneously^(14–16)^. Thus, although homeless populations represent a socially vulnerable whole/group, each part (subject) of this group must be apprehended and welcomed in its uniqueness and based on its personal and collective meaning of life.

Like other systems, health is subsidized by subsystems and, at the same time, it is part of a larger system that interacts with other social systems. Health as a complementary system is fed back from dialogical movements between users, professionals, services, communities that, in turn, interfere in the healthy living of individuals. Change in a subsystem interferes with the evolutionary dynamics of the larger system and vice versa^(15,16)^.

The quality and dynamics of the health system are determined, based on this approach, by the quality of relationships, interactions and dialogical and prospective associations with the different actors involved in the health care process^(27)^. A study reinforces this thinking by mentioning that health indicators are determined by the reception, ambience and bond with those users – homeless people – apparently more distant and invisible in the eyes of society^(28)^.

Conceiving health as a social good necessarily implies expanding the concept of being human, of citizenship, of living healthy. If the health professional has the skills to promote health as a social-common good, he also has the skills to evolve and prospect strategies that include different social classes, above all, homeless people^(29)^.

Complex thinking “questions the predictable, the absolute and the linearity of social phenomena”^(17)^. Achieving systemic-complex processes in the health context implies, therefore, promoting the interactivity and complementarity of those apparently disjunctive and disruptive phenomena.

The present study enables, based on the above, advances related to the expanded apprehension of the health phenomenon, that is, as a subsystem of a larger system – the social system. Another advance is related to the promotion of a new way of thinking and acting among health professionals, capable of overcoming the simplifying and reductionist paradigm, which conceives knowledge from a fragmented and disciplinary perspective, with a view to recognizing human beings in the context of their relationships social.

The proposition of only one theoretical framework – complex thinking for the basis of the discussions constitutes a limitation, although other bibliographic sources have been used. In short, theoretical and practical advances are intended, in the sense of leveraging social and health policies that consider the unique needs of homeless people. Just as the concept of citizenship cannot be reduced to the possibility of having rights and duties, in the same way healthy living cannot be reduced to biological human needs.

## FINAL CONSIDERATIONS

Health is conceived as a subsystem of the social system, based on a reflective framework that transcends any linear and punctual diagnostic perspective. Theoretical reflection on the social and health vulnerability of homeless people sparks a singular and multidimensional apprehension of the human being – a complex unit, par excellence, and which demands equally complex interventions.

Expanding the understanding of the human being – a street person, requires dialogical and prospective strategies on the part of the different social actors. It is based on the principle that the nursing professional, through health care, has the potential to promote ruptures and lead advances, based on references that enable new ways of intervening and promoting healthy living.
